# 

**Published:** 1899-06

**Authors:** 


					JUNE, 1899.
THE
AMERICAN JOURNAL
?w O F w?
DENTAL SCIENCE.
EDITED BY
F. J. S. GORGAS;-_
RICHARD dRA
Vol. 33.
No. ?.
BALTIMORE:
SNOWDEN &. COWMAN MFG. CO , 9 W. Fayette St.
LONDON:
TRUBNER A, CO., 60 Pate?no?ter Row.
Entered at the Post Office at Baltimore, Md., as Second-class Matter.
PK.YKBLE IN RDVHNCE.
IPUBLISHED MONTHLY
$2.50 PER HNNUM.

				

